# Application of 3D bioprinting technology apply to assessing Dangguiniantongtang (DGNT) decoctions in arthritis

**DOI:** 10.1186/s13020-024-00948-4

**Published:** 2024-07-08

**Authors:** Zhichao Liang, Yunxi Han, Tao Chen, Jinwu Wang, Kaili Lin, Luying Yuan, Xuefei Li, Hao Xu, Tengteng Wang, Yang Liu, Lianbo Xiao, Qianqian Liang

**Affiliations:** 1 Longhua Hospital, Shanghai University of Traditional Chinese Medicine, 725 Wan-Ping South Road, Shanghai, 200032 China; 2 Guanghua Hospital Affiliated to Shanghai University of Traditional Chinese Medicine, 540 Xinhua Road, Shanghai, 200052 China; 3 Spine Institute, Shanghai University of Traditional Chinese Medicine, 725 Wan-Ping South Road, Shanghai, 200032 China; 4 Key Laboratory of Theory and Therapy of Muscles and Bones, Ministry of Education (Shanghai University of Traditional Chinese Medicine), 1200 Cailun Road, Shanghai, 201203 China; 5 Institute of Arthritis Research in Integrative Medicine, Shanghai Academy of Traditional Chinese Medicine, 540 Xinhua Road, Shanghai, 200052 China; 6 Shanghai Key Laboratory of Orthopaedic Implant, Department of Orthopaedic Surgery, Shanghai Ninth People’s Hospital Affiliated Shanghai Jiao Tong University School of Medicine, 639 Zhizaoju Rd, Shanghai, 200011 China; 7 Institute of Rehabilitation Medicine, School of Rehabilitation Science, Shanghai University of Traditional Chinese Medicine, Engineering Research Center of Traditional Chinese Medicine Intelligent Rehabilitation, Ministry of Education, 1200 Cailun Road, Shanghai, 201203 China; 8 Department of Oral & Cranio-Maxillofacial Surgery, Shanghai Ninth People’s Hospital, College of Stomatology, Shanghai Jiao Tong University School of Medicine, 639 Zhizaoju Rd, Shanghai, 200011 China

**Keywords:** 3D bioprinting, Chondrocytes, Osteoblasts, Arthritis, Decoction

## Abstract

The aim of this study was to develop a three-dimensional (3D) cell model in order to evaluate the effectiveness of a traditional Chinese medicine decoction in the treatment of arthritis. Chondrocytes (ATDC5) and osteoblasts (MC3T3-E1) were 3D printed separately using methacryloyl gelatin (GelMA) hydrogel bioinks to mimic the natural 3D cell environment. Both cell types showed good biocompatibility in GelMA. Lipopolysaccharide (LPS) was added to the cell models to create inflammation models, which resulted in increased expression of inflammatory factors IL-1β, TNF-α, iNOS, and IL-6, and decreased expression of cell functional genes such as Collagen II (COLII), transcription factor SOX-9 (Sox9), Aggrecan, alkaline phosphatase (ALP), RUNX family transcription factor 2 (Runx2), Collagen I (COLI), Osteopontin (OPN), and bone morphogenetic protein-2 (BMP-2). The created inflammation model was then used to evaluate the effectiveness of Dangguiniantongtang (DGNT) decoctions. The results showed that DGNT reduced the expression of inflammatory factors and increased the expression of functional genes in the cell model. In summary, this study established a 3D cell model to assess the effectiveness of traditional Chinese medicine (TCM) decoctions, characterized the gene expression profile of the inflammatory state model, and provided a practical reference for future research on TCM efficacy evaluation for arthritis treatment.

## Introduction

Arthritis is an inflammatory disease that affects the joints in the limbs and the surrounding tissues. It is a major source of pain, disability, and socioeconomic costs globally. In patients with arthritis, various tissues are stimulated by inflammation and secrete high levels of inflammatory factors and matrix metalloproteinases (MMPs). This leads to massive matrix loss and excessive apoptosis of chondrocytes and osteoblasts, ultimately resulting in joint destruction and deformity. Nonsteroidal anti-inflammatory drugs (NSAIDs) and glucocorticoids are widely recognized as the primary pharmacological interventions for the treatment of arthritis [1]. The NSAIDs have the propensity to induce adverse reactions within the digestive, cardiovascular, and renal systems [2]. Furthermore, prolonged administration of glucocorticoids is associated with an increased susceptibility to hypertension, osteoporosis, and various other metabolic disorders [3, 4]. Despite the extensive clinical use of Dangguiniantong (DGNT) decoctions in China for the treatment of arthritis for thousands of years, there is limited scientific research to validate their therapeutic efficacy.

Relying solely on the conventional two-dimensional (2D) cell culture model for drug screening has shown limitations in achieving satisfactory in vivo and clinical efficacy for the screened drugs. This approach has been the prevailing method for a significant period of time, but its effectiveness has been questioned. Therefore, it is necessary to explore alternative models for drug screening. The scientific community is increasingly emphasizing and acknowledging the crucial role of the extracellular matrix (ECM) in governing cell behavior [5]. In order to achieve a more accurate representation of the conditions present within living organisms, extensive advances have been made in the field of three-dimensional (3D) cell culture techniques. 3D cultures offer a more accurate and in-depth portrayal of organismal conditions in vitro by faithfully replicating the intercell and ECM signaling microenvironment [6, 7].

This disparity is evident at the gene and protein expression levels [8]. 3D models are now extensively utilized in the study of various diseases, including lung cancer [9], breast cancer [10], retinal glial cells [11] and skeletal muscle [12]. However, the availability of 3D models specific to joints is still relatively limited. Currently, most studies of 3D printed joint scaffold models focus on tissue engineering for articular cartilage repair [13–16]. There are very few in vitro models targeting the assessment of drug efficacy in arthritis [17, 18]. In vitro models for efficacy studies of herbal decoction are not yet available.

ATDC5 and MC3T3-E1 cells are widely used in arthritis research. Many studies have shown that LPS can induce inflammatory factor expression in ATDC5 cells [19–21], and induces the down-regulation of functional genes in MC3T3-E1 cells [22, 23]. This study aimed to establish a 3D cell model using methacryloyl gelatin (GelMA) that accurately replicates the inflammatory conditions of ATDC5/MC3T3-E1 cells. The pioneering model was developed to evaluate the therapeutic efficacy of Traditional Chinese medicine (TCM) decoctions in the treatment of arthritis. Initially, the biocompatibility of the model used in this study was assessed, followed by the observation of gene expression in ATDC5/MC3T3-E1 cells under inflammatory conditions. Subsequently, the model was used as a platform to evaluate the anti-inflammatory properties of DGNT decoctions. The findings collectively confirmed the 3D model that effectively validates the effectiveness of TCM decoctions in treating arthritis.

## Materials and methods

### Cells culture

The ATDC5 and MC3T3-E1 cell lines were obtained from the prestigious Cell Bank of the Chinese Academy of Sciences (Shanghai, China). The complete medium for ATDC5 cell line comprised DMEM/F12 supplemented with 10% fetal bovine serum and 1% penicillin–streptomycin solution. The complete culture medium required for the MC3T3-E1 cell line consisted of α-MEM supplemented with 10% fetal bovine serum and 1% Penicillin–streptomycin solution.

### Bioink

The materials GelMA and Lithium phenyl-2,4,6-trimethylbenzoylphosphinate (LAP) were procured from EFL-Tech Co., Ltd. (Suzhou, China). 5% (w/v) of GelMA and 0.25% (w/v) of LAP were solubilized in PBS to prepare the hydrogel solution. The solution thus obtained was sterilized in a 60 °C oven for one hour and later stored in a 37 °C incubator. Subsequently, ATDC5/MC3T3-E1 cells were added to the prepared hydrogel solution at a concentration of 2 × 10^6^/ml. This mixture constituted the bioink used in this study.

### The preparation process of the 3D cell model

The 3D printing of the cell model was carried out using a 3D printer manufactured by Regenovo Biotechnology Co. Ltd. Detailed parameters for the printing programs are provided as follows: temperature control set to 20 ℃, model layer thickness of 0.3 mm, with a total number of 4 layers. The height of each individual layer was set to 1.2 mm, while the total height of the model measured 2 mm, width measured 6.5 mm × 6.5 mm. Following the printing process, the bioink was exposed to UV light for a duration of 10–15 s to achieve instant solidification.

### CCK-8 assays

To evaluate cell proliferation, the CCK-8 assay (Dojindo, Shanghai, China) was performed using the respective CCK-8 kit. Following the culture of 3D cell constructs for assorted durations in 48-well plates, the CCK-8 assay solution was introduced, and the resultant enzymatic marker was assessed for its corresponding OD value.

### Live/dead staining

The 3D cell constructs were seeded into 48-well plates. Staining reagents were added on day 1, day 2, day 4, day 7 and day 10 according to kit instructions (Sigma), and cells growth was observed using a fluorescent microscope.

### Detection of alkaline phosphatase (ALP) activity

The ALP staining kit was purchased from Shanghai Beyotime Biotechnology Co., Ltd. Cells or 3D cell models were treated with Western and IP cell lysates (3D printed models were homogenized by adding grinding beads). Add the samples to 96-well plates and measure the absorbance following the instructions provided in the kit.

### Drug administration

The herbal medicines used to prepare the DGNT decoctions were obtained from the Longhua Hospital, which is affiliated with the Shanghai University of Traditional Chinese Medicine. The herb dosage was converted using a conversion factor of 9.01. The concentration of the herb after the decoctions was determined to be 1.89 g/ml. The preparation of the DGNT decoctions involved 15 types of herbs, as shown in Table 1. To prepare the DGNT decoctions lyophilized powder, the prepared decoctions were centrifuged, and the resulting supernatant was filtered and then stored at −80 °C. It was further lyophilized in a vacuum dryer for 48–72 h and stored at −80 °C. For the in vitro experiments, the lyophilized powder was dissolved in complete medium, filtered using a 0.22 μm filter, and subsequently used.

**Table 1:** The composition of DGNT decoctions

Latin name	Chinese name of medicine	Dosage, g
Notopterygium incisum	Qiang Huo	15
Rhizoma Cimicifugae	Sheng Ma	3
Atractylodes macrocephala	Bai Shu	3
Angelica sinensis (Oliv.) Diels	Dang Gui	9
Glycyrrhiza uralensis Fisch	Gan Cao	15
Scutellaria baicalensis Georgi	Huang Qin	3
ArtemisiacapillarisThunb	Yin Chen	15
Anemarrhena asphodeloides Bunge	Zhi Mu	9
Saposhnikovia divaricata	Fang Feng	9
Puerariae Lobatae Radix	Ge Gen	6
Atractylodes lan cea	Cang Shu	9
Panax ginseng C. A. Mey	Ren Shen	6
Sophora flavescens	Ku Shen	6
Alisma plantago-aquatica Linn	Ze Xie	9
Polyporus	Zhu Ling	9

### Quantitative RT-PCR

Cells were harvested, and cDNA was generated using Trizol (Invitrogen) and PrimeScriptTM RT reagent Kit (cat. #RR037A). The gene expression was quantified by RT-PCR using QuantiTect SYBR Green (HieffTM). The primers for qPCR are shown in Table 2. All primers were purified and synthesized by the Huada Company (HuaDa, Shenzhen, China).

**Table 2:** Primer sequence for RT-PCR

Gene	5′to 3′
COLI	F: CTTTGCTTCCCAGATGTCCT
	R: CGGTGTCCCTTCATTCCAG
COLII	F: ACCTTGGACGCCATGAAA
	R: CAGGGCAGTGTATGTGAACC
IL-6	F: TGCCTTCTTGGGACTGAT
	R: TTGCCATTGCACAACTCTTT
iNOS	F: GAGCGAGTTGTGGATTGTC
	R: CCAGGAAGTAGGTGAGGG
MMP13	F: GCAGTTCCAAAGGCTACA
	R:CTCGGAGACTGGTAATGG
Sox9	F: AGTACCCGCATCTGCACAAC
	R: ACGAAGGGTCTCTTCTCGCT
IL-1β	F: CTGGTACATCAGCACCTCAC
	R:AGAAACAGTCCAGCCCATAC
TNF-α	F: AGTGACAAGCCTGTAGCCC
	R: GAGGTTGACTTTCTCCTGGTAT
ALP	F: AACAACCTGACTGACCCTTC
	R: ATCCTGCCTCCTTCCACTA
Runx2	F: AACTTCCTGTGCTCCGTGCTG
	R: TCGTTGAACCTGGCTACTTGG
Osterix	F: AGGAGGCACAAAGAAGCCATACG
	R:ATGCCTGCCTTGTACCACGAGC
OCN	F:GGACCATCTTTCTGCTCACTCTG
	R: GTTCACTACCTTATTGCCCTCCTG
OPN	F: CCAGCAGCAGGACTGAAGGAGC R: TTCACCGGGAGACAGGAGGC
BMP-2	F: CAACACCGTTCAGCTTCC
	R: TTCCCACTCATTTCTTTCC
Aggrecan	F: GACTGTCTATCTACACGCCAACCA
	R: GATGTCGTCTTCACCACCCAC
β-Actin	F: GGTGGGAATGGGTCAGAAGG
	R: GTTGGCCTTAGGGTTCAGGG

F, forward; R, reverse

### Statistical analysis

Statistical analyses were performed with GraphPad Prism 9.0 software. Data are presented as mean ± SEM. Analyses between 2 groups used unpaired Student t-test. When comparing the difference among > 2 groups, one-way ANOVA analysis of variance was used followed by a Tukey multiple comparisons posttest. *P* values < 0.05 were considered statistically significant.

## Results

### The design of the 3D cell model

**Fig. 1:**
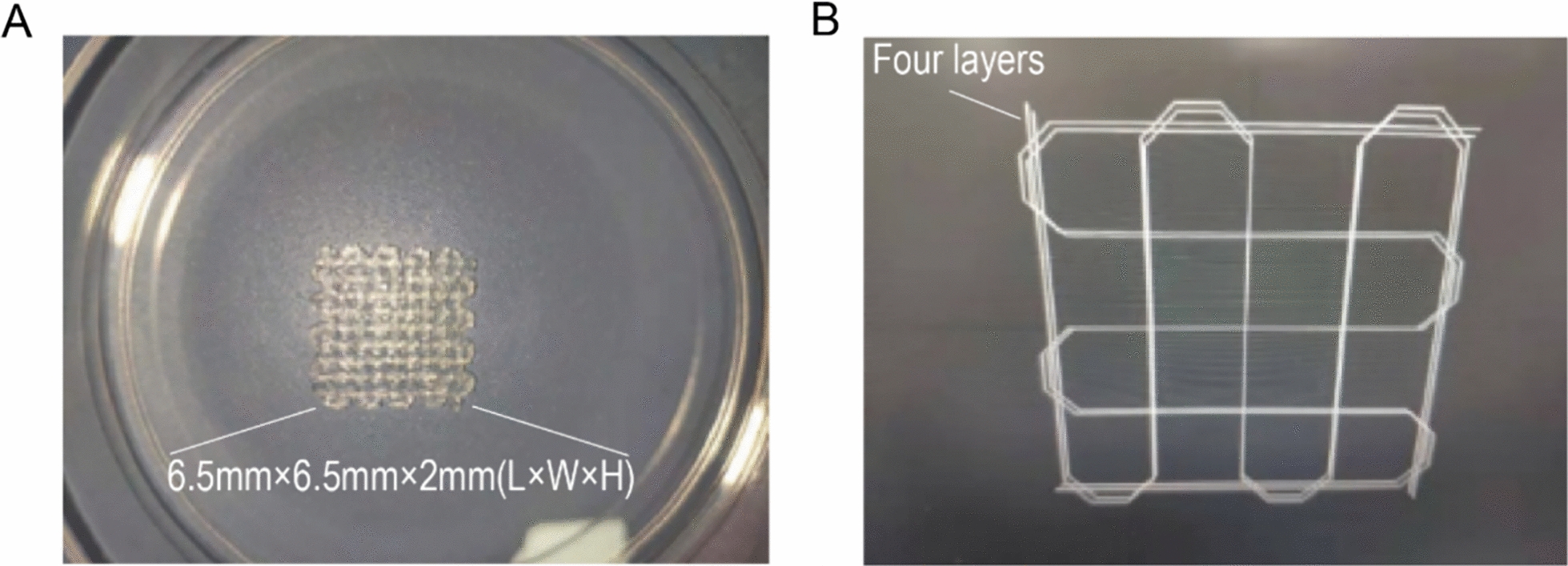
The design of a 3D cell model. **A** 3D printed cell model samples. The height of each individual layer was set to 1.2 mm, while the total height of the model measured 2 mm, width measured 6.5 mm × 6.5 mm. **B** 3D printing design path of the scaffol. The printing programs are provided as follows: temperature control set to 20 ℃, model layer thickness of 0.3 mm, with a total number of 4 layers

Regenovo Bio-3D printers offer design pathways and printing functions, guaranteeing reasonable porosity and consistent printing performance (Fig. 1).

### The excellent biological characterization of the 3D cell model

**Fig. 2:**
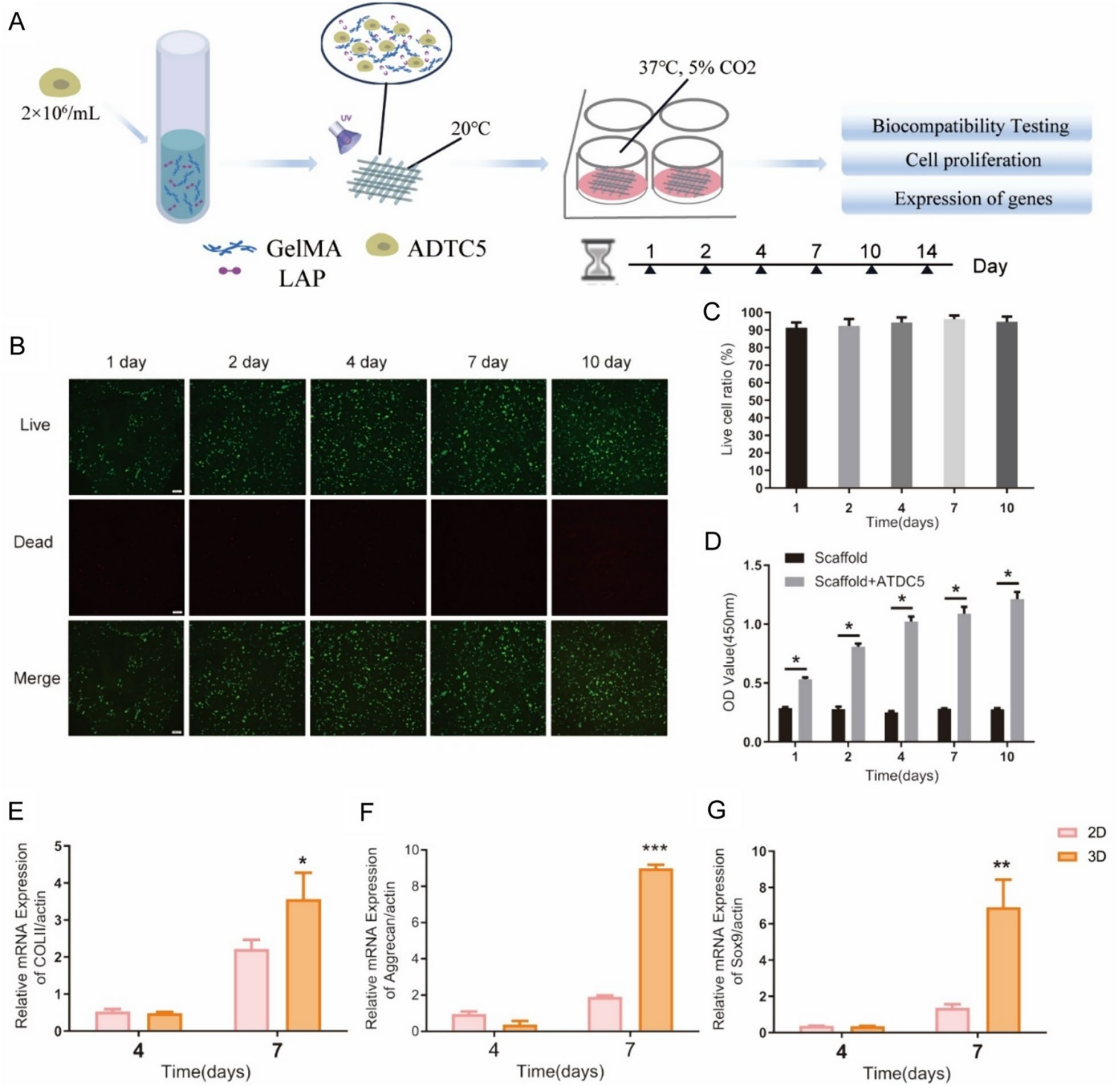
Biological characterization of the ATDC5 cell model. **A** Schematic diagram of the experimental. **B** ATDC5 Cell were stained using the LIVE/DEAD Cell Viability/Cytotoxicity Assay Kit, with green fluorescence indicating live cells and red fluorescence indicating dead cells. **C** The survival rate of cell in 3D cell model after various days of culture is shown (n = 6). **D** Cell proliferation was analyzed using the CCK-8 assay (n = 5, t-test, *P < 0.05). **E**–**G** RT-PCR assay was performed to analyze the mRNA expression of COLII, Sox9 and Aggrecan in ATDC5 cells (n = 5, t-test, *P < 0.05, **P < 0.01, ***P < 0.001 VS. 2D group)

The schematic diagram showed in Fig. 2A depicts the construction process of the ATDC5 cells model and the corresponding biological studies. ATDC5-containing hydrogel scaffolds were cultured for 1, 2, 4, 7, and 10 days, followed by live/dead cells staining. Analysis of the results demonstrated that the survival rate of ATDC5 cells within the 3D model exceeded 90% (Fig. 2B, C). The cell activity assay using CCK-8 was conducted, and the hydrogel scaffolds containing ATDC5 demonstrated notable proliferative activity compared to the blank scaffold group (Fig. 2D). It is important to identify and analyze specific marker genes in order to understand the functional traits and properties of chondrocytes. In contrast to the conventional 2D culture of ATDC5 cells, our 3D cell model exhibited a significant upregulation in the expression of the functional gene Collagen II (COLII) mRNA, with a 1.5-fold increase on day 7 (Fig. 2E). Moreover, the expression of Aggrecan mRNA exhibited a remarkable 4.5-fold increase on day 7 (Fig. 2F), while the expression of transcription factor SOX-9 (Sox9) mRNA demonstrated an even greater increase of fivefold (Fig. 2G).

**Fig. 3:**
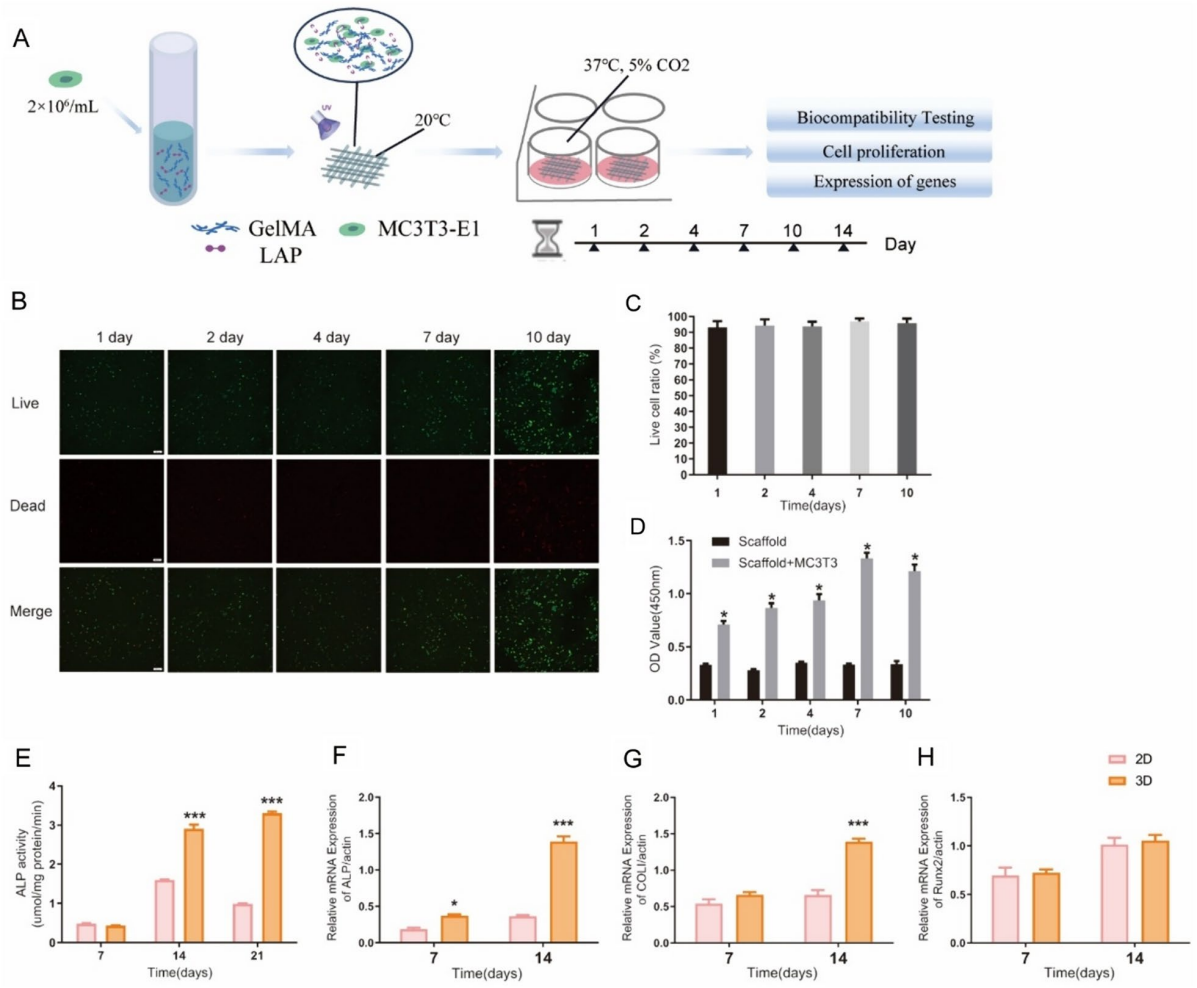
Biological characterization of the MC3T3-E1 cell model. **A** Schematic diagram of the experimental. **B** MC3T3-E1 Cell were stained using the LIVE/DEAD Cell Viability/Cytotoxicity Assay Kit, with green fluorescence indicating live cells and red fluorescence indicating dead cells. **C** The survival rate of cells in 3D cell model after various days of culture is shown (n = 6). **D** Cell proliferation was analyzed using the CCK-8 assay (n = 5, t-test, *P < 0.05). **E** Alkaline phosphatase activity assay of MC3T3-E1 cells (n = 5, t-test, ***P < 0.001 VS. 2D group). **F**–**H** RT-PCR assay was performed to analyze the mRNA expression of ALP, COLI and Runx2 in MC3T3-E1 cells (n = 5, t-test, *P < 0.05, ***P < 0.001 VS. 2D group)

The schematic diagram showed in Fig. 3A depicts the construction process of the MC3T3-E1 cell model and the corresponding biological studies. MC3T3-E1-containing hydrogel scaffolds were cultured for varying durations of 1, 2, 4, 7, and 10 days. Afterward, they were subjected to live/dead cell staining. Analysis of the obtained results revealed that the survival rate of MC3T3-E1 cells within the 3D model surpassed 90%, as depicted in Fig. 3B–C. The CCK-8 assay was performed to assess cell activity, where the hydrogel scaffolds containing MC3T3-E1 cells exhibited a significant increase in proliferative activity compared to the blank scaffold group, as illustrated in Fig. 3D. Quantification of ALP activity in MC3T3-E1 cells revealed a noteworthy increase within the 3D model compared to the conventional 2D culture. Specifically, ALP activity within the 3D model was twofold higher on day 14 and reached a threefold increase on day 21, as depicted in Fig. 3E. RT-PCR analysis of MC3T3-E1 cells revealed distinct differences in gene expression profiles between the 3D model and the conventional 2D culture. In the MC3T3-E1 cell 3D model, ALP mRNA expression was twofold higher than in the 2D culture on day 7 and nearly fourfold higher on day 14. Similarly, the expression of Collagen I (COLI) mRNA showed a twofold increase on day 14. However, there was no significant difference in the expression of RUNX family transcription factor 2(Runx2) mRNA compared to the control 2D culture (Fig. 3F–H).

### Construction of 3D cell model in LPS-induced inflammatory state

**Fig. 4:**
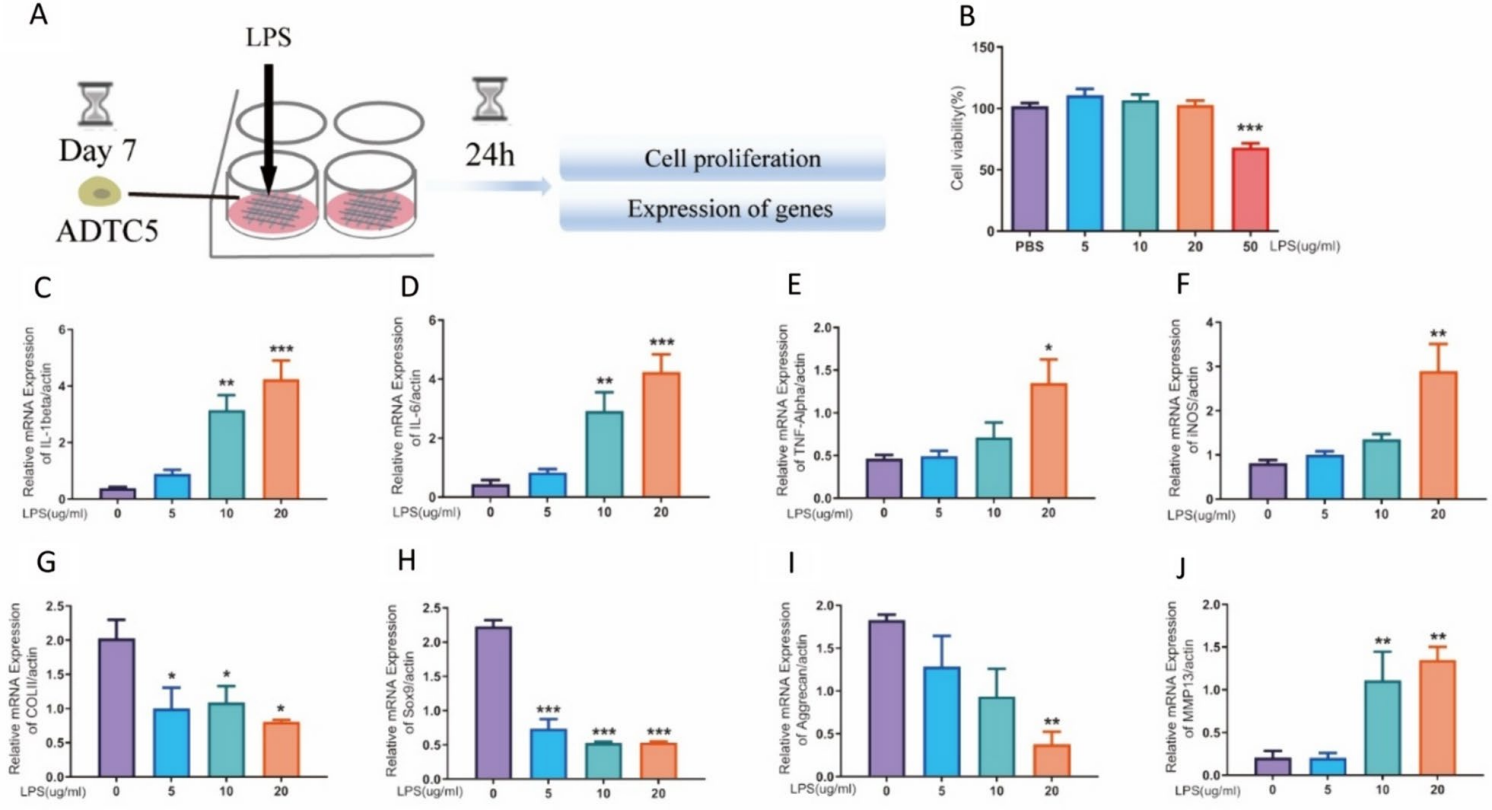
The expression of genes associated with the ATDC5 3D cell model was changed by distinct concentrations of lipopolysaccharide (LPS). **A** Schematic diagram of the experimental. **B** Effect of LPS on the cell activity of ATDC5 cells (n = 5, one way ANOVA, ***P < 0.001 VS. PBS group). **C**–**F** The mRNA expression of inflammatory factors (IL-1β, IL-6, TNF-α, and iNOS) under the LPS-induced inflammatory state (n = 3, one way ANOVA, *P < 0.05, **P < 0.01, ***P < 0.001 VS. PBS group). (G-J) The mRNA expression of functional genes (COLII, Sox9, Aggrecan and MMP13) under the LPS-induced inflammatory state (n = 3, one-way ANOVA, *P < 0.05, **P < 0.01, ***P < 0.001 VS. PBS group)

The process of stimulating the 3D cell model (ATDC5) with lipopolysaccharide (LPS) is depicted in Fig. 4A. The CCK-8 assay was used to evaluate the impact of LPS on cell activity within a 3D model of ATDC5 cells. The results showed significant inhibition of activity at a concentration of 50 μg/ml (Fig. 4B). The investigation further revealed that LPS stimulation negatively influenced the gene expression of ATDC5 cells. The analysis of RT-qPCR indicates a significant upregulation in the expression of inflammatory mediators such as IL-1β, IL-6, TNF-α, and iNOS after LPS treatment in the 3D model of ATDC5 cells (Fig. 4C–F). Concurrently, a substantial decrease was observed in the expression of COLII, Aggrecan, and Sox9 (Fig. 4G–I). Moreover, LPS stimulation elicited an increased expression of matrix metallopeptidase 13 (MMP13) (Fig. 4J).

**Fig. 5:**
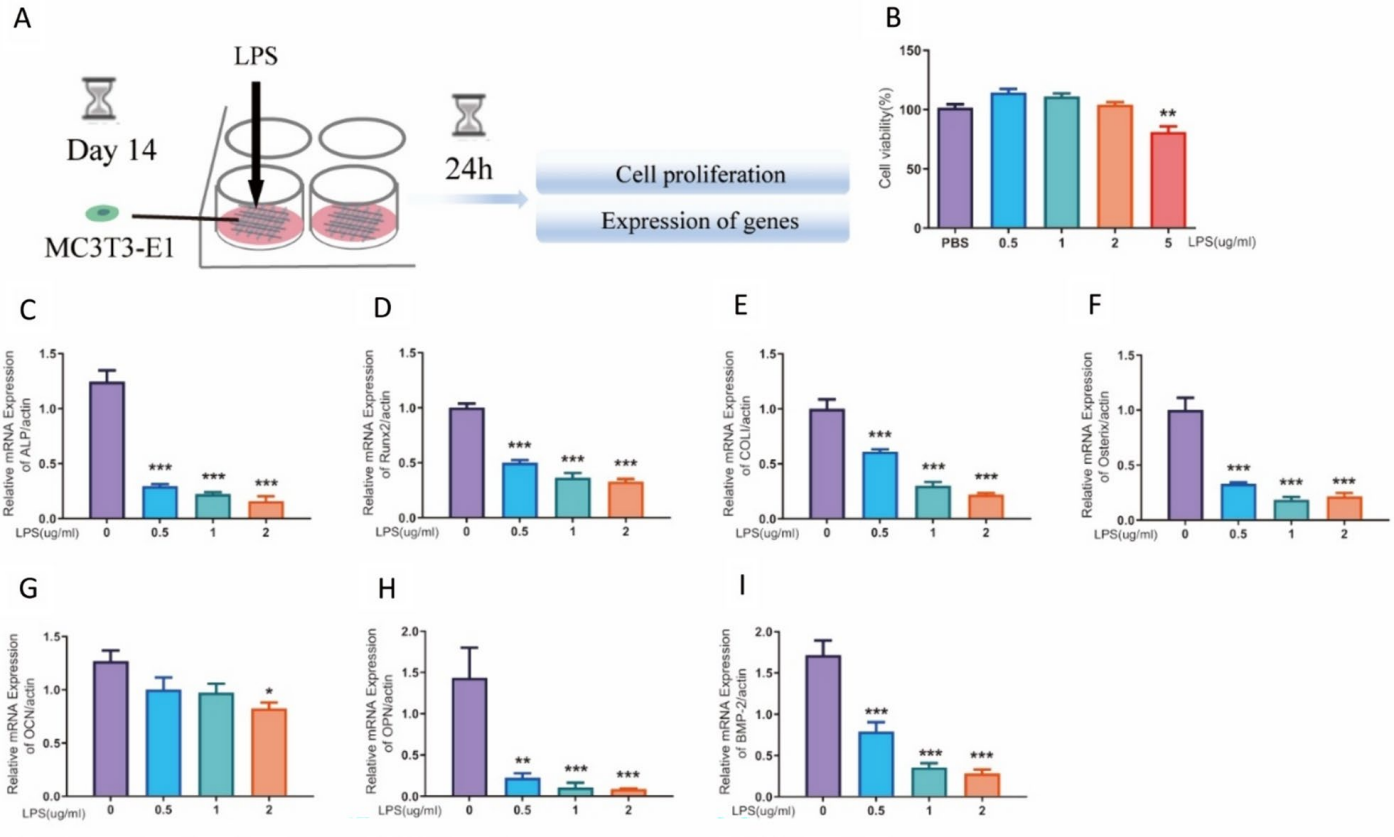
The expression of genes associated with the MC3T3-E1 3D cell model was changed by distinct concentrations of LPS.** A** Schematic diagram of the experimental. **B** Effect of LPS on the cell activity of MC3T3-E1 cells (n = 5, one way ANOVA, **P < 0.01 VS. PBS group). **C**–**I** The mRNA expression of functional genes (ALP, Runx2, COLI, Osterix, OCN, OPN, and BMP-2) under the LPS-induced inflammatory state. (n = 3, one-way ANOVA, *P < 0.05, **P < 0.01, ***P < 0.001 VS. PBS group)

The process of stimulating the 3D cell model (MC3T3-E1) with lipopolysaccharide (LPS) is depicted in Fig. 5A. The CCK-8 assay showed that LPS inhibited cell activity in the 3D model of MC3T3-E1 cells (Fig. 5B) at a 5 μg/ml concentration. In the 3D model of MC3T3-E1 cells, LPS stimulation resulted in decreased expression of functional genes ALP, Runx2, COLI, Osterix, Osteocalcin (OCN), Osteopontin (OPN), and bone morphogenetic protein-2(BMP-2) (Fig. 5C–I).

### DGNT attenuates inflammation and promotes bone formation in cell models of inflammatory states

**Fig. 6:**
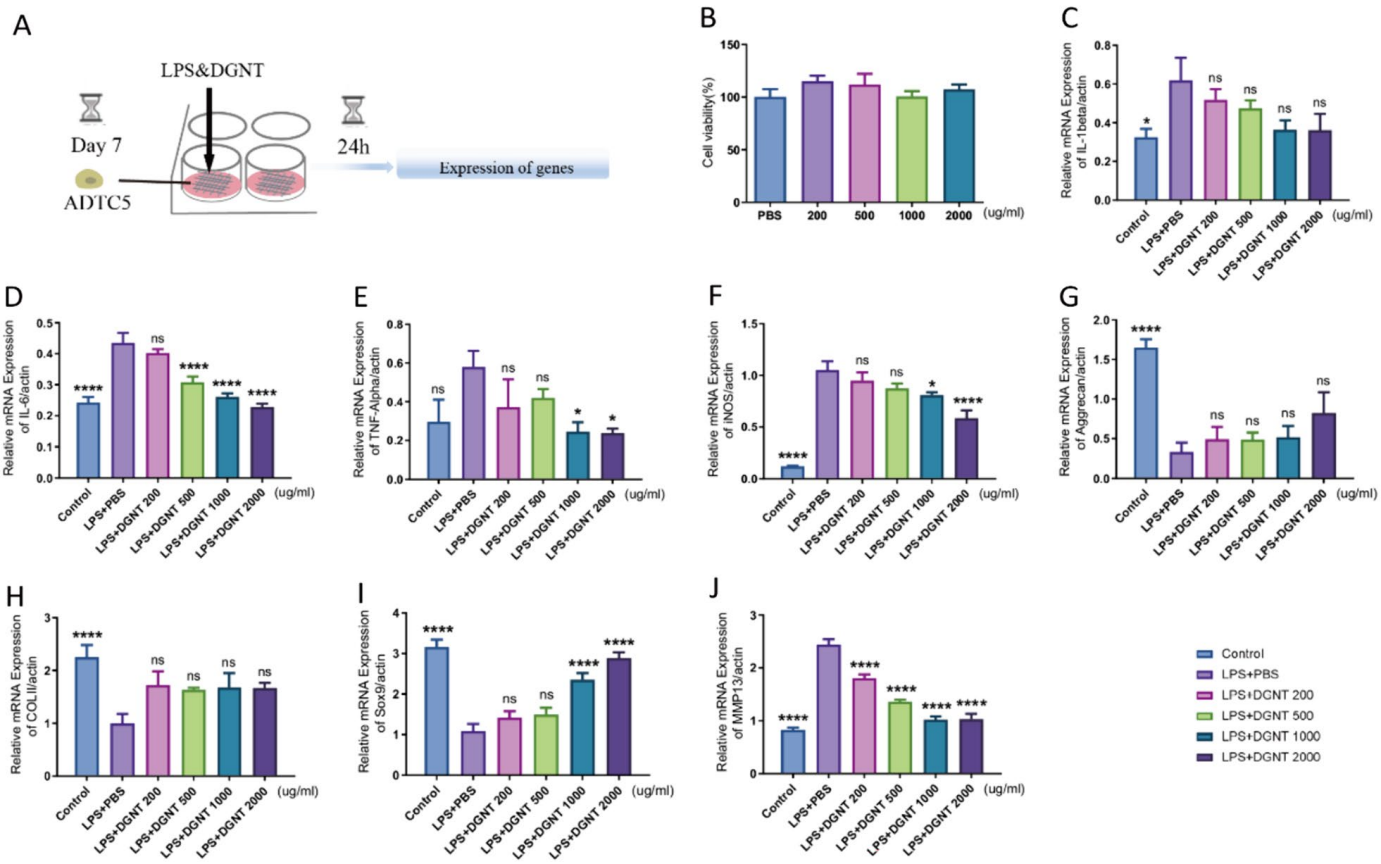
DGNT has reduced inflammation caused by LPS in ADTC5 cell and increased the expression of relevant functional genes.** A** Schematic diagram of the experimental**. B** Effect of DGNT on the activity of ATDC5 cell models. **C**–**F** LPS-induced mRNA expression of inflammatory factors (IL-1β, IL-6, TNF-α, and iNOS) in response to DGNT intervention (n = 10, one way ANOVA, *P＜0.05, ****P < 0.0001 VS. LPS + PBS group). **G**–**J** LPS-induced mRNA expression of functional genes (Aggrecan, COLII, Sox9 and MMP13) in the inflammatory state under DGNT intervention (n = 10, one-way ANOVA, ****P < 0.0001 VS. LPS + PBS group)

The experimental procedure of DGNT affecting 3D cell models (ATDC5) in inflammatory states was depicted in a schematic diagram (Fig. 6A). The cytotoxicity of the drug was assessed using the ATDC5 cell model. The ATDC5 cells were treated with DGNT for 24 h, and cell viability was evaluated using CCK-8. It was observed that DGNT at concentrations of 200 μg/ml, 500 μg/ml, 1000 μg/ml and 2000 μg/ml had minimal effect on the ATDC5 cell model (Fig. 6B). A concentration-dependent inhibitory effect on IL-1β, IL-6, TNF-α, and iNOS in the 3D model of ATDC5 cell was observed in the RT-qPCR results, indicating the suppressing inflammation properties of the DGNT (Fig. 6C–F). Furthermore, the expression of functional genes, including Aggrecan, COLII, and Sox9, were upregulated, while the expression of MMP13 was downregulated in response to the DGNT treatment (Fig. 6G–J).

**Fig. 7:**
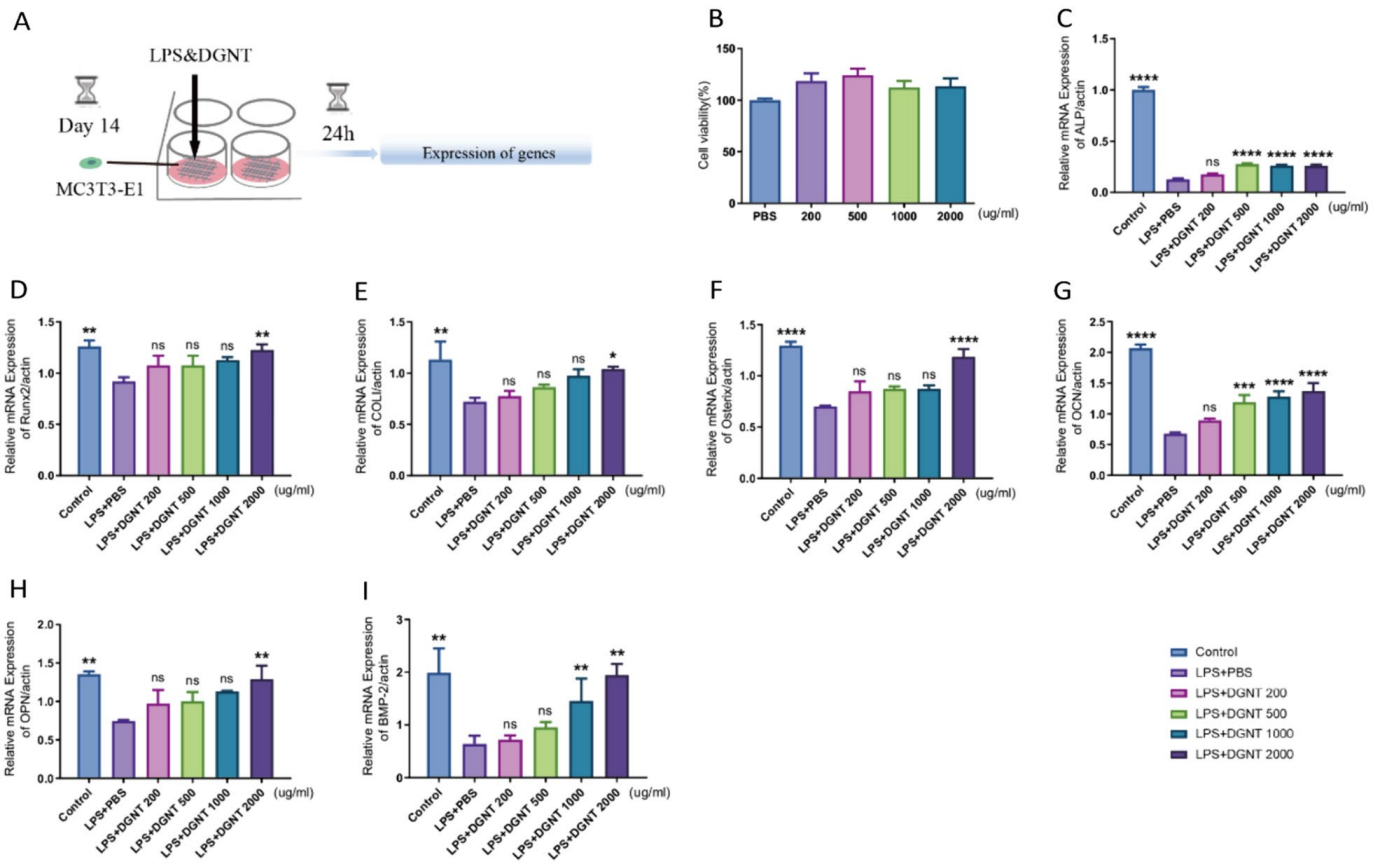
DGNT enhanced functional gene expression in MC3TE-E1 cell under LPS stimulation. **A** Schematic diagram of the experimental. **B** Effect of DGNT on the activity of MC3T3-E1 cell model. **C**–**I** mRNA expression analysis of essential functional genes (ALP, Runx2, COLI, Osterix, OCN, OPN and BMP-2) under LPS-induced inflammatory conditions, with intervention by DGNT (n = 10, one-way ANOVA, *P < 0.05, **P < 0.01, ***P < 0.001, ****P＜0.0001 VS. LPS + PBS group)

The experimental procedure of DGNT affecting 3D cell models (MC3T3-E1) in inflammatory states was depicted in a schematic diagram (Fig. 7A). The 3D model of MC3T3-E1 cell was intervened with DGNT for 24 h, and cell viability was assessed using the CCK8 assay. It was observed that DGNT, at concentrations of 200 μg/ml, 500 μg/ml, 1000 μg/ml, and 2000 μg/ml, had a negligible impact on the cell activity of the MC3T3-E1 3D model (Fig. 7B). RT-PCR analysis revealed that DGNT concentration-dependently enhanced the expression of ALP, Runx2, COLI, Osterix, OCN, OPN, and BMP-2 (Fig. 7C–I).

## Discussion

In this study, a 3D cell model of ATDC5/MC3T3-E1 cells and an inflammation model were constructed. We constructed a preliminary 3D cell model. On this model we can use LPS to induce a simple inflammatory osteoblast/chondrocyte model. The various types of arthritis have different characteristics, they all show a pathological process of inflammatory infiltration of bone and cartilage during their development. Rather than looking at a specific type of arthritis, we have focused on bone and cartilage damage. By comparison, it was found that the expression of inflammatory factors in ATDC5 cells was up-regulated in the model, which is consistent with existing studies. The down-regulation of functional genes, such as COLII, Runx2, and ALP in ATDC5/MC3T3-E1 cells is consistent with previous studies [24–27]. We found that the normal 3D model exhibited excellent biological effects in the absence of LPS stimulation. In 3D cellular inflammation models, LPS needs to be used at double or even higher doses to achieve the desired inflammation induction [19, 28–33]. The cause of the discrepancy may be related to the bioink, but we prefer to attribute it to the spatial distribution of cells. DGNT decoctions reduced the expression of inflammatory factors in vitro, which is consistent with previous efficacy studies of DGNT decoctions [34, 35]. The ability of DGNT decoctions to enhance the expression of functional genes aligns with the findings of other studies on drugs targeting genes that enhance osteoblast function [36–38]. The experimental results suggest that these 3D cell models can be used to assess the efficacy of TCM decoctions.

In conventional 2D cell culture models, cells are restricted to adhering to the substrate in contact with the culture surface [5, 39]. Hydrogel scaffolds provide a 3D structure that more closely resembles in vivo tissues [40, 41], in 3D culture, cells are capable of adhering to the culture surface in a more widespread manner. 3D culture helps maintain the morphology and function of osteoblasts/chondrocytes [42]. The degree of cells adhesion and stretching can impact crucial functions related to proliferation, apoptosis, and differentiation. The morphology of cells growth becomes more diverse in 3D model culture and resembles the diversity in vivo tissues [39, 43, 44]. Additionally, physiological interactions between cells and the ECM are replicated in a 3D model culture, which more accurately mimics the growth environment within the body [45, 46]. Compared to 2D cell culture, 3D model culture better simulate signaling between cells. This is important for studying cellular molecular pathways and signaling involved in the development of arthritis.

Although the application of 3D cell model to arthritis drug screening is in its early stages, several studies have researched their potential advantages. Researchers chose chondrocytes [47–50], synovial fibroblasts [51], RA fibroblast-like-synoviocytes [52] and vascular endothelial cells [51] as the research subjects in the constructed models, based on the pathological characteristics of different types of arthritis. Of particular interest is the fact that the vast majority of researchers chose to simulate the 3D state by culturing the cells in a hydrogel matrix without using 3D printing technology. Compared to cells cultured within a 3D matrix gel system, implementing 3D printed scaffolds offers a clear advantage in providing cells with a spatial structure. The strategies employed by the researchers to induce inflammation varied in their approaches to modeling inflammatory activation. Y [47] and Satyavrata Samavedi [49] chose to use co-culture with macrophages activated by LPS as an inflammatory activation condition. However, Lin [51] and Rosser [48] used cytokines such as TNF-α, Li [50] used IL-1β. The utility in assessing the efficacy of herbal decoctions, which has not been reported in other studies. It is worth mentioning that the 3D model is easy to construct, has a low construction cost, and can be reproduced easily. This makes it, which provides a valuable and practical reference for research in this field. In most studies of arthritis, the TNF-α gene is overexpressed under inflammatory conditions [21, 53, 54]. In the 3D cellular inflammation model of this study, ATDC5 cells had elevated TNF-a gene expression (Fig. 4E).

While the current study has provided significant findings, it is essential to acknowledge certain limitations. Notably, LPS, a constituent of the outer membranes of gram-negative bacteria, can induce inflammation and impair the functioning of osteoblasts and chondrocytes in cell cultures [23, 55, 56]. Therefore, we have prioritized using LPS as the stimulus, acknowledging its singular but essential role. However, it is worth noting that future investigations could also explore alternative stimulation modalities, such as TNF-α or IL-1β. Due to the disparities in the culture conditions utilized for ATDC5 cells and MC3T3-E1 cells, it was not feasible to achieve a mixed culture of these cell types under inflammation-inducing conditions. Consequently, we were unable to establish a hybrid model with our current efforts. However, this will be a crucial aspect of our future research endeavors.

## Conclusion

In this study, our utilization of 3D printing technology allowed us to create a model of joint inflammation, which enabled us to objectively assess the potential therapeutic effects of herbal decoctions. Our results revealed that the ATDC5/MC3T3-E1 cells exhibited enhanced viability and functionality when seeded in scaffolds, as opposed to conventional 2D cultures. Moreover, our study revealed the anti-inflammatory effects demonstrated by the DGNT in our inflammation model. Crucially, our research holds essential implications as it provides a valuable reference and establishes the foundation for integrating 3D printing technology in investigating the therapeutic effectiveness associated with traditional Chinese medicine decoctions.

## Data Availability

All data related to this study can be obtained from the authors on reasonable request.

## Abbreviations

ALP: Alkaline phosphatase
BMP-2: Bone morphogenetic protein-2
COLI: Collagen I
COLII: Collagen II
DGNT: Dangguiniantongtang
ECM: Extracellular matrix
GelMA: Methacryloyl gelatin
LPS: Lipopolysaccharide
LAP: Lithium phenyl-2,4,6-trimethylbenzoylphosphinate
MMPs: Matrix metalloproteinases
MMP13: Matrix metallopeptidase 13
NSAIDs: Nonsteroidal anti-inflammatory drugs
OCN: Osteocalcin
OPN: Osteopontin
Runx2: RUNX Family Transcription Factor 2
Sox9: Transcription factor SOX-9
TCM: Traditional Chinese medicine
3D: Three-dimensional
2D: Two-dimensional
